# Culture, group membership, and face recognition. Commentary: Will you remember me? Cultural differences in own-group face recognition biases

**DOI:** 10.3389/fpsyg.2016.01101

**Published:** 2016-07-19

**Authors:** Kai Kaspar

**Affiliations:** Department of Psychology, University of CologneCologne, Germany

**Keywords:** cross-cultural comparison, group membership, face recognition, ingroup bias, prioritization

Literature reports that same-race faces are better recognized than cross-race faces. This cross-race effect has been observed, inter alia, in European Americans (MacLin et al., 2004) as well as in Asian Americans and East Asians (Michel et al., 2006; Hayward et al., 2008). Although research showed that cross-race effects reflect a superiority in the processing of components and configurations of own-race faces (Hayward et al., 2008), empirical evidence showed that (arbitrary) social categorization similarly contributes to an ingroup bias in face recognition (MacLin and Malpass, 2001). This finding suggests that mere social categorization is sufficient to bias face recognition performance in favor of ingroup faces, reflecting a motivational preference to individuate ingroup members (Hugenberg et al., 2010).

Following these findings, Ng et al. (2016) examined whether participants' cultural background moderates the effect of arbitrarily determined social group membership on the recognition performance for ingroup/outgroup targets displaying neutral facial expressions. According to their focal hypothesis, North Americans should preferentially define their ingroups on broader social categories, whereas East Asians should define their ingroups with a focus on preexisting relationships established through friendship and family (Brewer and Yuki, 2007). Therefore, Ng and colleagues expected that European Canadians, but not East Asians, would show an ingroup bias in face recognition when group membership is arbitrarily determined by a minimal group manipulation: A color-coding system indicated whether one's own personality fits the personality of a displayed person (Study 1) or whether one's preexisting university affiliation fits that of a displayed person (Study 2). Same colors indicated ingroup faces, different colors indicated outgroup faces. In both studies, European Canadians recognized previously observed ingroup faces better than outgroup faces. East Asian Canadians did not show this bias for arbitrarily determined ingroup faces. Hence, the authors concluded that culture moderates the effect of mere social categorization on face recognition. Overall, these studies provide a valuable extension of previous research by introducing a cross-cultural perspective. However, some crucial aspects should be considered in future research in this field.

## The empirical aspect

The result pattern for Asian Canadians showed some inconsistency across studies. European Canadians recognized White faces better than East Asian faces in both studies. In contrast, Asian Canadians showed a better memory for Asian faces in Study 1 (personality), whereas they better recognized White faces in Study 2 (university affiliation), suggesting a culture-specific effect of group membership. Generally, when observing and memorizing a sequence of many faces, one's cognitive capacities are quickly exceeded, so one has to prioritize the targets. In the absence of other veridical information about a target's relevance we usually refer to contextual information to reduce situational uncertainty (Kaspar, 2013; Kaspar and Krull, 2013) and ambiguity (Kaspar, in press). Correspondingly, color as a contextual sign for one's university affiliation might have helped to identify and separate targets of high (ingroup) and low (outgroup) priority in European Canadians, reflected in a better recognition of ingroup faces. In contrast, social categorization in terms of university affiliation might have primed thoughts of acculturation processes in Asian Canadians instead. Given the central argument of motivationally driven prioritization of visual input (Hugenberg et al., 2010), it is conceivable that the first-generation Asian Canadians tested by Ng and colleagues have been sensitized toward acculturation processes, leading to an increased motivation to accurately process White faces representing the host culture's population. In contrast, when color indicated the personality type (Study 1), such cognitive processes were rather unlikely and hence cross-race effects were visible in both European and Asian Canadians. Consequently, in order to verify the validity of the reported results, the study should be replicated with Asians living in a respective country without a personal involvement in acculturation processes. More generally, it should be considered that test-taking behavior can be influenced by participants' relation to the culture in which the test is administered (Cofresi and Gorman, 2004).

## The methodological aspects

**Figure 1:**
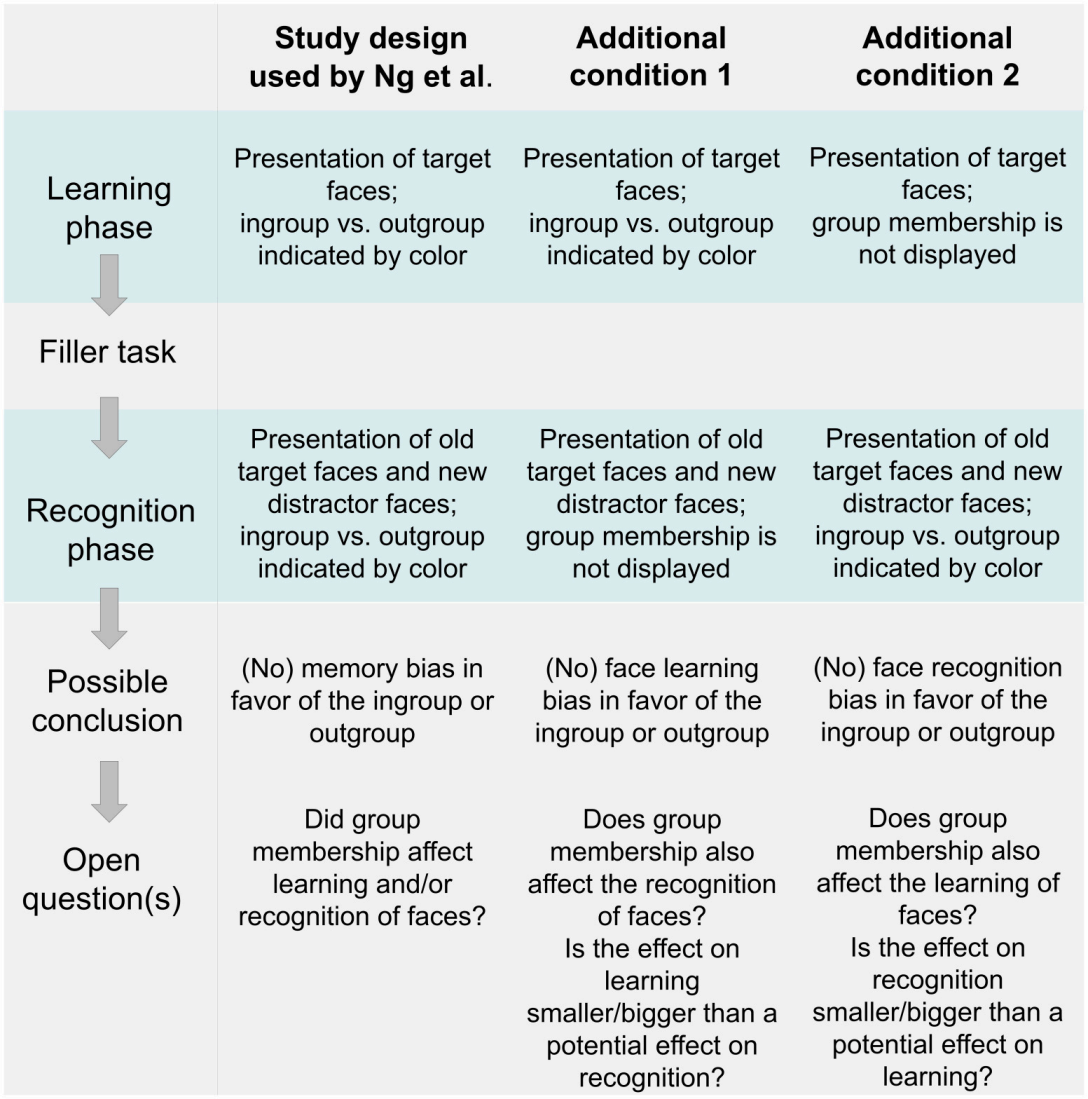
**The traditional study design used by Ng et al. (2016) and two additional experimental conditions proposed for future studies**. In condition 1 group membership is exclusively indicated in the learning phase, whereas in condition 2 group membership is introduced only in the recognition phase.

The study by Ng and colleagues shares one methodological limitation with several other studies investigating group effects on face recognition performance, because it remains open whether group membership affects cognitive processes in the learning or/and recognition phase. In addition to the judgmental responses investigated by Ng et al., other research suggests that the recognition bias for ingroup faces reflects a motivationally driven top-down modulation of cognitive processes, leading to an increased activity of the fusiform face area in the recognition phase (Van Bavel et al., 2011). Thus, group membership might affect selective attention and cognitive processing in terms of encoding face attributes when observing target faces in the learning phase. Alternatively, group membership might (also) increase the motivation to thoroughly compare memory content with current visual input in the recognition phase. To compare these alternatives, future studies should extend the traditional study design used by Ng et al. by two additional conditions, as illustrated in Figure 1.

Furthermore, it is important to pay attention when selecting face stimuli for cross-cultural comparisons. Previous studies revealed that attractive faces can intensify approach motivation (Zebrowitz et al., 2015) and that face recognition performance may depend on the emotion displayed (Di Domenico et al., 2015; Righi et al., 2015). Given cultural differences in the judgment of facial attractiveness (Sorokowski et al., 2013) and in the interpretation of emotional facial expressions (Yuki et al., 2007), the attractiveness and emotional valence of same-race and other-race faces have to be validated by all investigated culture groups to prevent potential confounds.

## The conceptual aspect

Finally, let us assume the validity of Ng et al.'s focal hypothesis stating that Asians preferentially define their ingroups by preexisting relationships established through friendship and family. If so, it might be that we will nonetheless find ingroup favoritism regarding face recognition in Asians when a minimal group manipulation is applied to a set of faces sampled from the pool of the participant's family or group of friends. Indeed, such a “second order” effect appears to be an inherent part of the assumption that ingroup membership is initially determined by personal relationships in (some) Asian cultures, while this does not make the prioritization of multiple targets superfluous in the face of strongly limited cognitive capacities.

## Author contributions

The author confirms being the sole contributor of this work and approved it for publication.

### Conflict of interest statement

The author declares that the research was conducted in the absence of any commercial or financial relationships that could be construed as a potential conflict of interest.

## References

[B1] Brewer M. B. Yuki M. (2007). Culture and social identity, in Handbook of Cultural Psychology, eds Kitayama S. Cohen D. (New York, NY: Guilford Press), 307–322.

[B2] Cofresi N. I. Gorman A. A. (2004). Testing and assessment issues with Spanish-English bilingual Latinos. J. Couns. Dev. 82, 99–106. 10.1002/j.1556-6678.2004.tb00290.x

[B3] Di Domenico A. Palumbo R. Mammarella N. Fairfield B. (2015). Aging and emotional expressions: is there a positivity bias during dynamic emotion recognition? Front. Psychol. 6:1130. 10.3389/fpsyg.2015.01130 26300822 PMC4523706

[B4] Hayward W. G. Rhodes G. Schwaninger A. (2008). An own-race advantage for components as well as configurations in face recognition. Cognition 106, 1017–1027. 10.1016/j.cognition.2007.04.002 17524388

[B5] Hugenberg K. Young S. G. Bernstein M. J. Sacco D. F. (2010). The categorization-individuation model: an integrative account of the other-race recognition deficit. Psychol. Rev. 117, 1168–1187. 10.1037/a0020463 20822290

[B6] Kaspar K. (2013). Embodied cognition is a weighty matter: heaviness influences the perception of disease severity, drug effectiveness, and side effects. PLoS ONE 8:e78307. 10.1371/journal.pone.0078307 24244302 PMC3823885

[B7] Kaspar K. (in press). Arousal-biased preferences for sensory input: an agent-based multisource perspective. Behav. Brain Sci. 10.1017/S0140525X15001855 28347388

[B8] Kaspar K. Krull J. (2013). Incidental haptic stimulation in the context of flirt behavior. J. Nonverbal Behav. 37, 165–173. 10.1007/s10919-013-0154-0

[B9] MacLin O. H. Malpass R. S. (2001). Racial categorization of faces: the ambiguous race face effect. Psychol. Public Pol. L. 7, 98–118. 10.1037/1076-8971.7.1.98

[B10] MacLin O. H. Van Sickler B. R. MacLin M. K. Li A. (2004). A re-examination of the cross-race effect: the role of race, inversion, and basketball trivia. N. Am. J. Psychol. 6, 189–204.

[B11] Michel C. Rossion B. Han J. Chung C. S. Caldara R. (2006). Holistic processing is finely tuned for faces of one's own race. Psychol. Sci. 17, 608–615. 10.1111/j.1467-9280.2006.01752.x 16866747

[B12] Ng A. H. Steele J. R. Sasaki J. Y. (2016). Will you remember me? Cultural differences in own-group face recognition biases. J. Exp. Soc. Psychol. 64, 21–26. 10.1016/j.jesp.2016.01.003

[B13] Righi S. Gronchi G. Marzi T. Rebai M. Viggiano M. P. (2015). You are that smiling guy I met at the party! Socially positive signals foster memory for identities and contexts. Acta Psychol. 159, 1–7. 10.1016/j.actpsy.2015.05.001 26000956

[B14] Sorokowski P. Kościński K. Sorokowska A. (2013). Is beauty in the eye of the beholder but ugliness culturally universal? Facial preferences of Polish and Yali (Papua) people. Evol. Psychol. 11, 907–925. 10.1177/147470491301100414

[B15] Van Bavel J. J. Packer D. J. Cunningham W. A. (2011). Modulation of the fusiform face area following minimal exposure to motivationally relevant faces: evidence of in-group enhancement (not out-group disregard). J. Cogn. Neurosci. 23, 3343–3354. 10.1162/jocn_a_00016 21452952

[B16] Yuki M. Maddux W. W. Masuda T. (2007). Are the windows to the soul the same in the East and West? Cultural differences in using the eyes and mouth as cues to recognize emotions in Japan and the United States. J. Exp. Soc. Psychol. 43, 303–311. 10.1016/j.jesp.2006.02.004

[B17] Zebrowitz L. A. Franklin R. G. Palumbo R. (2015). Ailing voters advance attractive congressional candidates. Evol. Psychol. 13, 16–28. 10.1177/147470491501300102 25562113 PMC4353482

